# How are hospitals in England, Scotland and Wales caring for women with nausea and vomiting in pregnancy: a national service evaluation

**DOI:** 10.1186/s12913-025-12909-0

**Published:** 2025-08-25

**Authors:** Melanie Nana, Natalie Suff, Maria Gregori, Catherine Nelson-Piercy, Catherine Williamson

**Affiliations:** 1 Department of Obstetric Medicine, Guy’s and St Thomas’ NHS Foundation Trust, Westminster Bridge Road, London, SE1 7EH England; 2 Department of Women and Children’s Health, King’s College London, The Strand, London, WC2RLS England; 3 Department of Metabolism, Digestion and Reproduction, Imperial College London, South Kensington, London, SW72BX England

**Keywords:** Nausea and vomiting of pregnancy, Hyperemesis gravidarum, Guideline-recommended care

## Abstract

**Background:**

Nausea and vomiting of pregnancy (NVP) affects up to 90% of pregnant women but many struggle to access guideline-recommended care. Following a King’s Policy Institute policy laboratory, arranged to explore barriers to care, it was recommended that a scoping review of current national practice was carried out. This study aims to describe NVP services in England, Scotland and Wales and compare management to national guidance.

**Methods:**

An online survey was distributed to all 139 maternity units in England, Scotland and Wales using freedom of information services. Data were downloaded onto an Excel spreadsheet and statistical analysis performed using GraphPad Prism 10.

**Results:**

Responses were received from 129/139 hospitals giving a response rate of 92.8%. Routine screening for NVP/HG at a woman’s booking visit is offered in 37/129 (28.7%) of the hospitals. Treatment in the community was offered in 19/129 (14.8%) and ambulatory management available in 108/129 (83.7%) of hospitals that responded. As per RCOG guidance only 60/129 (47%) of hospitals correctly prescribe a combination of recommended first, second and third-line antiemetics and whether the maternity unit is secondary or tertiary, or whether patients are primarily managed in an obstetric or gynaecology setting, does not influence provision of guideline-recommended care, (secondary 39/85 (45.8%) vs. tertiary 21/44 (47.7%) *p* = 0.84 and obstetric 12/34 (35.3%) vs. gynaecology setting 48/95 (50.5%) *p* = 0.13, respectively). A proton pump inhibitor was prescribed in 64/129 (49.6%) of units and thiamine for patients with persistent vomiting in 90/129 (69.8%). Guideline-recommend intravenous fluid management (0.9% normal saline) is used in 93/129 (72.1%) of units. In those where it is not, 5/36 (13.9%) use dextrose solution (recognised to precipitate Wernicke’s encephalopathy). Routine mental health screening occurs in 54/129 (41.9%) of units. Pre-pregnancy counselling is offered to women with a history of severe NVP/HG planning a future pregnancy in 22/129 (17.1%) of units.

**Conclusions:**

Significant variation in HG care exists across England, Scotland and Wales. Despite guidance published by the RCOG the treatment women currently receive is not routinely evidence-based and in some cases has potential to cause harm.

**Supplementary Information:**

The online version contains supplementary material available at 10.1186/s12913-025-12909-0.

## Background

Nausea and vomiting of pregnancy (NVP) affects up to 90% of pregnant women and represents the leading cause of hospital admission in the first trimester of pregnancy [1]. Annual costs to the National Health Service attributed to NVP have been estimated to be up to £62 million as a consequence of hospital admissions, ambulance call-outs and visits to primary care practitioners [1–3]. Hyperemesis Gravidarum (HG) is a severe form of NVP affecting 3.6% of the pregnant population [4]. While symptoms of NVP typically resolve by 16 weeks’ gestation, 20% of those with HG will suffer symptoms throughout their entire pregnancy [5, 6].

If left untreated HG can result in life-threatening complications for the mother including fatal cardiac arrythmia, thiamine deficiency leading to Wernicke’s encephalopathy and Vitamin K deficiency resulting in coagulopathy [7]. The condition can also result in psychological morbidity with up to 5% of sufferers terminating a wanted pregnancy because of the condition and 7% experiencing regular suicidal ideation [8, 9]. In a study of 5018 women with HG we demonstrated that difficulty accessing appropriate care and subsequently being bed-bound by the condition, unable to look after existing children or being unable to remain in employment are risk factors for these adverse outcomes [8, 10].

The Royal College of Obstetricians and Gynaecologists Green-top guidelines (GTG) [11, 12] recommend that where possible women with NVP/HG be managed in the community/primary care to avoid unnecessary hospital admissions and disruption to a woman’s life. When these community measures fail, ambulatory day care should be used. Inpatient management should be provided for those who are unable to keep down oral antiemetics, develop signs of dehydration or weight loss despite oral treatment and those with comorbidity. The guideline outlines recommended first, second- and third-line antiemetic therapy and promotes use of proton pump inhibitors in women also symptomatic of gastroesophageal reflux disease. Inpatient treatment will often require intravenous fluid hydration and in rare cases enteral/parenteral nutrition. An assessment of mental health is recommended due to the known consequences of this condition on mental health.

In February 2023 a King’s Policy Institute London policy laboratory was held to bring together key stakeholders to address the reasons why, despite comprehensive evidenced-based guidance [12], women in the UK do not universally access appropriate care. A scenario which is not only likely to have significant consequences for the women but also cost implications for the NHS, with untreated patients re-attending hospital services and presenting with the complications of the disease. A key recommendation from the policy laboratory was to gain an understanding of the landscape of care nationally and current compliance with national guidance. The urgency to address inequalities in HG care in the UK was also discussed in a parliamentary debate focussed on ‘Hyperemesis Gravidarum Awareness’ in the House of Commons [13].

This study describes current services for NVP and HG in England, Scotland and Wales and compares management to recommendations in the Royal College of Obstetricians and Gynaecologists green-top guideline [12].

## Methods

An online survey comprising 12 questions (S1) was designed by the authors to include details of each maternity unit’s NVP/HG service design, management protocol and whether the unit provided pre-pregnancy counselling for women with a history of HG. The survey contained a mixture of multiple choice and free text questions. The survey was distributed to all 139 maternity units in England, Scotland and Wales in July 2023 using freedom of information services. After six weeks a reminder email was sent to all units that had not replied. A further reminder was sent in October 2023 and we closed responses in March 2024. As freedom of information services were used we did not seek ethical approval.

The data were downloaded onto an Excel spreadsheet. Free text questions where numerical answers were given were standardised. Data were summarised using descriptive summary statistics, with results reported as numbers (percentages). Sites were separated into secondary or tertiary care units depending on the Department of Health intensive care unit level care. Comparisons of categorial variables between the two units were performed using the χ^2^ test. A *P*-value of < 0.05 was considered statistically significant. GraphPad Prism 10 (GraphPad Software, San Diego, CA, USA) was used for statistical analysis.

## Results

**Fig. 1:**
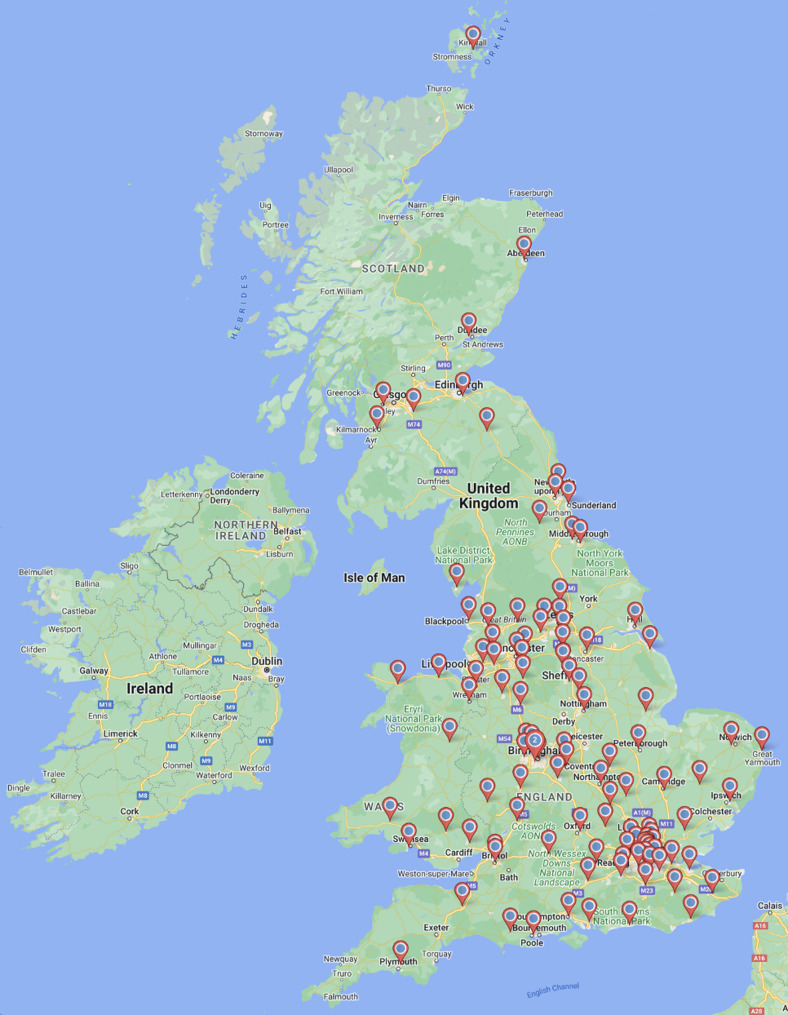
Distribution of hospitals that responded to the survey

Responses were received from 129/139 hospitals giving a response rate of 92.8%. In total, 85/129 (65.9%) were secondary care and 44/129 (34.1%) were tertiary centre units. The geographical distribution of the responding hospitals is presented in Fig. 1.

### Screening for NVP/HG, community care and ambulatory management

Routine screening for NVP/HG at a woman’s booking visit is offered in 37/129 (28.7%) of the hospitals that responded. Treatment in the community is offered in 19/128 (14.8%); ambulatory management in a hospital setting is available in 108/129 (83.7%) hospitals. Patients are more likely to be offered ambulatory care if they are managed in a tertiary unit when compared to a secondary unit (secondary 67/85 (79%]) vs. tertiary 41/44 (93%), *p* = 0.03). Further details of the community and ambulatory services are outlined in S2.

### Inpatient management

#### Admission criteria and location

**Fig. 2:**
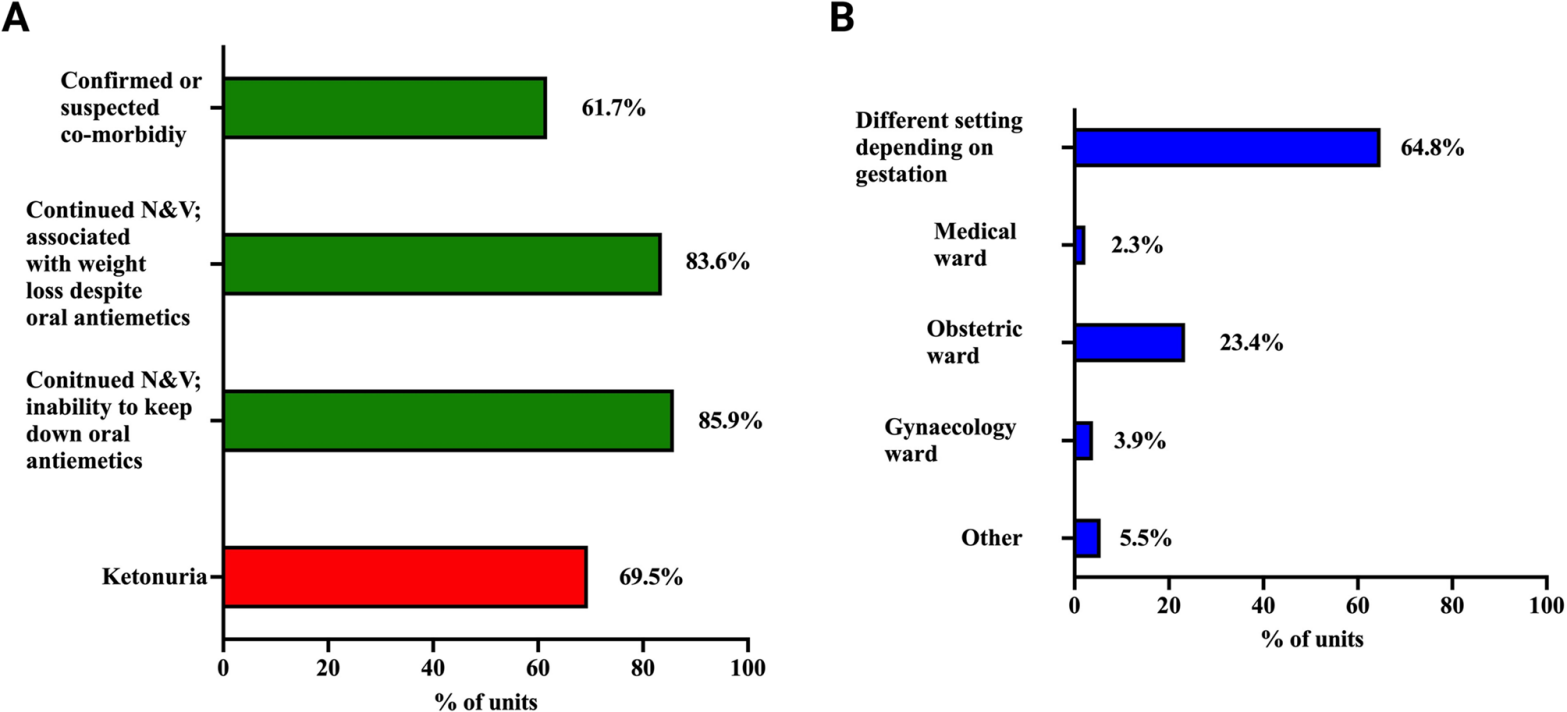
**a** Proportion of hospitals using each admission indication **b** Locations of admitted patients

The proportion of hospitals using each of five admission criteria is shown in Fig. 2a. This multiple-choice question included three criteria recommend in the RCOG guideline (represented in green bars) and one that is discouraged in the RCOG guideline and would actively not be recommended (represented in red). Figure 2b shows the locations in which admitted patients were cared for; in those who selected different settings depending on gestation 17/81(21%) managed patients on a gynaecology ward up until 18 weeks of gestation and on an obstetric ward thereafter.

#### Management

In terms of treatment 41/129 (31.8%) hospitals routinely recommended a trial of ginger for the management of NVP/HG, 32/129 (24.8%) accustimulation and 5/129 (3.9%) hypnosis.

Each of the 129 responding units indicated which of the antiemetics recommended in the guideline they had available in their formulary for the management of NVP/HG and whether they prescribed each as a 1st, 2nd or 3rd line antiemetic (Table 1). In total, 9/129 (7.0%) of hospitals prescribing ondansetron required patients to sign a ‘risk form’ prior to prescription. One hospital prescribing cyclizine, one prescribing prochlorperazine, two prescribing ondansetron, 20/120 (16.7%) prescribing metoclopramide and two prescribing corticosteroids indicated that they prescribe them for a maximum of five days. Five hospitals prescribing corticosteroids prescribed them only after the first trimester of pregnancy. A proportion of each hospitals did not recommend any second or third line antiemetics.

**Table 1:** Proportion of hospitals prescribing each guideline-recommended antiemetic as a first, second or third line antiemetic therapy

	Hospital prescribing			
	1st line	2nd line	3rd line	PRN
Guideline recommended 1st line medications				
Cyclizine (*n* = 116)	109 (85.2%)	6 (4.7%)	1 (0.8%)	
Prochlorperazine (*n* = 117)	86 (67.2%)	28 (21.9%)	3 (2.3%)	
Promethazine (*n* = 76)	66 (51.6%)	8 (6.3%)	2 (1.6%)	1 (0.8%)
Chlorpromazine (*n* = 41)	26 (20.3%)	13 (10.2%)	2 (1.6%)	1 (0.8%)
Guideline recommended 2nd line medications				
Ondansetron (*n* = 90)	2 (1.6%)	54 (42.2%)	34 (26.6%)	
Metoclopramide (*n* = 98)	14 (10.9%)	71 (55.5%)	13 (10.2%)	2 (1.6%)
Domperidone (*n* = 54)	3 (2.3%)	44 (34.4%)	7 (5.5%)	3 (2.3%)
Guideline recommended 3rd line medications				
Corticosteroids (*n* = 72)	1 (0.8%)		71 (55.5%)	12 (9.4%)

*n* = the total number of hospitals prescribing the antiemetic; hospitals were able to select more than one answer

*Abbreviations*: *PRN* as needed

**Fig. 3:**
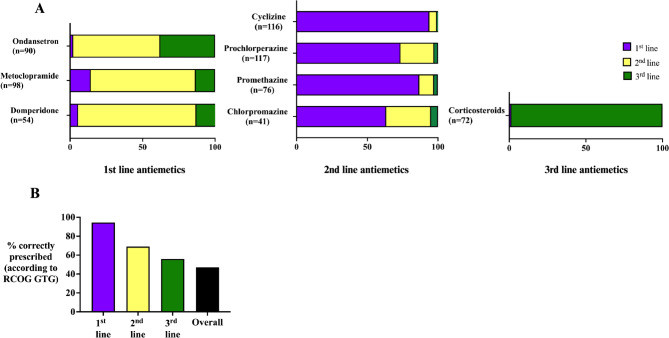
**a** Proportion of hospitals prescribing each guideline-recommended antiemetic **b** Proportion of hospitals correctly prescribing a combination of recommended antiemetics

Figure 3a demonstrates the proportion of hospitals prescribing each guideline-recommended antiemetic as a first, second- or third-line antiemetic therapy. As per RCOG guidance 60/129 (47%) of hospitals correctly prescribe a combination of recommended first, second and third-line antiemetics (Fig. 3b). Whether the maternity unit is secondary or tertiary, or whether patients are primarily managed in an obstetric or gynaecology setting, does not influence provision of guideline-recommended care, (secondary 39/85 (45.8%) vs. tertiary 21/44 (47.7%) *p* = 0.84 and obstetric 12/34 (35.3%) vs. gynaecology setting 48/95 (50.5%) *p* = 0.13, respectively)

A proton pump inhibitor was prescribed in 64/129 (49.6%) of units and thiamine for patients with persistent vomiting in 90/129 (69.8%). Pyridoxine is used in 29/129 (22.5%) of units and diazepam in 4/129 (3.1%); neither of these medications are recommended for use in the RCOG guideline.

Guideline-recommend intravenous fluid management (0.9% normal saline) is used in 93/129 (72.1%) of units. In those where it is not, 5/36 (13.9%) use dextrose solution (recognised to precipitate Wernicke’s encephalopathy). 32/129 (24.8%) units use Hartmann’s solution.

Enteral or parenteral nutrition for patients resistant to treatment is offered in 70/129 (54.3%) of units; tertiary units were more likely to offer it than secondary (tertiary 32/44 (72.7%) vs. secondary 38/85 (44.7%) *p* = 0.003). Routine mental health screening occurs in 54/129 (41.9%) of units. Pre-pregnancy counselling is offered to women with a history of severe NVP/HG planning a future pregnancy in 22/129 (17.1%) of units.

## Discussion

This service evaluation describes current services in England, Scotland and Wales available for women with NVP/HG. It demonstrates significant heterogeneity between different maternity units in terms of the location in which the women are managed and which antiemetic therapy they receive. Access to treatment beyond antiemetic therapy also varies, particularly with regard to mental health support and pre-pregnancy counselling.

Despite NVP being common, affecting up to 90% of the pregnant population [1] and HG being associated with significant physical and psychological morbidity and mortality [7, 8], only around one quarter of women are screened for these conditions at their booking visits. This is often a pregnant woman’s first encounter with a healthcare professional in pregnancy and represents an opportunity to screen for and initiate treatment for NVP/HG. We recognise that pregnant women have anxieties around taking medication in pregnancy [10], particularly antiemetics, and this would allow targeted counselling to enable women to make informed decisions about their treatment.

Community care, including treatment at home, is only accessible for 14.8% of units from whom responses were received. In a matched control study of 50 HG-women treated at home compared to 47 women managed in hospital, treatment at home was found to be safe, efficacious and significantly reduced the cost of care [14]. In a study involving in-depth interviews of women with the condition increased access to home treatment was recognised as a method to improve patient satisfaction [15]. Increased provision of services to deliver such care (e.g. hospital at home) is likely to improve patient experience while reducing inpatient costs; health economic analysis will be of value to guide this recommendation. Our study suggests that ambulatory management is now almost routine across the country with 84.4% of units offering it; this is consistent with RCOG guidance and has also been demonstrated to be effective, safe and in keeping with patient preference [16]. In a study at King’s College Hospital, London introduction of an ambulatory HG unit was found to reduce the length of stay per HG hospital admission, reduce the total number of inpatient nights per year and was associated with a substantial cost-saving of almost £100,000 per year [17].

RCOG recommended criteria for inpatient admission for management of HG include continued nausea and vomiting and inability to keep down oral antiemetics, continued nausea and vomiting associated with clinical dehydration or weight loss and presence of co-morbidity, due to the recognised complications in such cases [12]. While 85.9% and 83.6% of units, respectively admit patients for the first two of these indications only 61.7% admit patients due to the presence of co-morbidity. This is a concern as increasing numbers of women now enter pregnancy with a medical co-morbidity [18] and the presence of continued NVP/HG may pose significant risk, for example in cases of type 1 diabetes mellitus, or make it impossible to keep down critical medications for example anti-seizure medications or corticosteroids for patients with adrenal insufficiency. Historically ketonuria has been used as a marker of dehydration and an indicator of severity in patients with HG but this is not the case [19]. Ketonuria is a marker of starvation and therefore has no role in the assessment of dehydration or severity in HG; as such use of this biochemical test has been removed from the RCOG guidance. Clinical assessment and use of the Hyperemesis level prediction or Pregnancy-Unique Quantification of Emesis scores are better markers and are preferred [20, 21]. It is therefore concerning that, despite national recommendations, ketonuria is still being used as an indication for hospital admission in 69.5% of units.

With regards management, almost one third of hospitals promote the use of ginger for the management of NVP/HG. This is ill-advised as it is not effective for these conditions and in one study was found to increase rates of gastroesophageal reflux disease [22]. It too has been removed from the RCOG guidance.

For the majority of antiemetic therapies, where they were available they were used appropriately. However, less than half of the hospitals that responded prescribe a guideline-recommended first, second- and third-line medication suggesting variability in management across units. Notably, 26.6% of hospitals that prescribe ondansetron which is recognised to be one of the most effective antiemetics for the management of NVP/HG (and more effective than antihistamines and metoclopramide [23, 24]) reserve this as a third line medication. In 7% of hospitals, women being prescribed ondansetron are required to sign a ‘risk form’, this is likely a consequence of an European Medicine Agency warning against the use of ondansetron in the first trimester of pregnancy due to a possible increase in the rates of orofacial clefts and cardiac defects [25]. The UK Teratology Information Service published a systematic review which concluded that available literature do not provide evidence [26] of a causal association and on consideration of benefit and risk it is advocated as a second line antiemetic in the RCOG guideline. We believe it is important that patients are appropriately counselled about this potential risk, which relates to a possible attributable increased risk of orofacial clefts of 4 in 10,000 cases [27]. Another common misconception in the management of HG is that metoclopramide, a dopamine receptor antagonist, can only be prescribed for five days due to the risk of extrapyramidal side effects and oculogyric crises. In our study 16.7% of the hospitals that prescribe metoclopramide limit its use to five days. This is not in keeping with guidance as the risk of these adverse outcomes is extremely small (< 0.2%), is most typically seen in those receiving the drug intravenously and most commonly occurs within 72 h of administration [28]. Furthermore, as it is recognised that one in five women have symptoms of HG that continue throughout their entire pregnancy, women who are given only short courses of antiemetics are unlikely to receive significant benefit [6]. In a study of 241 general practitioners, 93% identified cyclizine as being a safe medication in pregnancy, but only 58% believed any of the remaining antiemetics to be safe in pregnancy, reflecting a lack of awareness of the safety of guideline-recommended treatments amongst primary care healthcare professionals [29]. It is our hope that the appendices included in the RCOG guidance (including those specifically aimed at primary care and early pregnancy assessment units) will increase awareness of appropriate evidenced-based therapies and encourage updating of local guidelines and protocols for the management of NVP/HG [12].

Gastroesophageal reflux is reported in 64% of HG patients and can be associated with rare complications including erosive oesophagitis, bleeding and strictures [6]. Less than half of units included a proton pump inhibitor as part of the management of HG patients, thus it is likely that a number of women are not benefitting from appropriate treatment of associated reflux symptoms.

There have been no studies to determine the most appropriate intravenous fluid for the management of NVP/HG. However, the RCOG guideline recommendation to use 0.9% normal saline with supplementary potassium replacement as required reflects the risk of HG patients typically becoming hypochloraemic, hyponatraemic and hypokalaemic due to persistent vomiting. This solution is used in just less than three quarters of all units. Concerningly dextrose solutions, which contain no sodium or potassium and are recognised to precipitate Wernicke’s encephalopathy, particularly if used in high concentrations before thiamine replacement, are currently the intravenous fluid management of choice in five of the hospitals.

While we found that the location in which HG patients are managed varied significantly from unit to unit (Fig. 1b) this did not influence the likelihood that women would receive guideline-recommended treatment. Whether a unit was secondary or tertiary, did, however influence access to enteral or parenteral nutrition. Notably access to routine mental health screening is low (41.9% of units). This is lower than is clinically indicated, considering the high rates of suicidal ideation and longer-term psychiatric impacts suffered by patients with this condition. Better recognition of the need for mental health support for sufferers of HG is vital in order to prevent psychological morbidity and mortality [8, 30–32].

The role of pre-pregnancy counselling for women with a history of NVP/HG considering a further pregnancy has not been confirmed. It is offered in 17.1% of units in England, Scotland and Wales. In a small study, pre-emptive management of HG with a combination of doxylamine and pyridoxine was found to reduce severity of symptoms and reduce the number of weeks of symptoms in the subsequent pregnancy compared to the previous [33]. Pre-pregnancy counselling offers the opportunity to discuss such management strategies and enable the clinician to empower the woman to seek appropriate care. Further work is necessary to establish the benefits of such counselling to determine whether recommendations to increase provision and access to pre pregnancy counselling across an increased number of units are appropriate.

### Strengths and limitations

A large number of responses were received from the maternity units across England, Scotland and Wales giving a response rate of 92.8%. We therefore achieved good geographical spread, across different socioeconomic areas with responses received from both secondary and tertiary units. The responses that we received were largely complete with minimal missing data.

It was outwith the scope of this service evaluation to determine the outcomes of women managed within the care settings or to determine satisfaction and this should be the focus of future work.

## Conclusion

Significant variation in HG care exists across England, Scotland and Wales. Despite guidance published by the RCOG the treatment women currently receive is not routinely evidence-based and in some cases has potential to cause harm. Future policy work should focus on the implementation of the national guidance/evidence-based approaches and education of those working within maternity care to ensure that local guidelines and practice are in line with best practice.

## Supplementary Information

Supplementary Material 1.

Supplementary Material 2.

## Data Availability

The datasets used and/or analysed during the current study are available from the corresponding author on reasonable request.

## Abbreviations

NVP: Nausea and vomiting of pregnancy
HG: Hyperemesis gravidarum
RCOG: Royal College of Obstetricians and Gynaecologists
GTG: Green-top guidelines
UK: United Kingdom
