# Supplementary material for: How are hospitals in England, Scotland and Wales caring for women with nausea and vomiting in pregnancy: a national service evaluation
Source: BMC Health Serv Res. 2025 Aug 25;25:1128. doi: 10.1186/s12913-025-12909-0 (PMC12376445; doi:10.1186/s12913-025-12909-0)
Supplement: Supplementary file 1 — Supplementary Material 1. [file 12913_2025_12909_MOESM1_ESM.docx]

Dear whom it may concern,

A recent policy lab was carried out at King’s College London aimed at advancing the Women’s Health Strategy by improving access to appropriate management of Hyperemesis Gravidarum (HG) for pregnant people who suffer with this condition.

Key stakeholders felt it would be valuable to gain an understanding of the way in which HG/ nausea and vomiting of pregnancy (NVP) care is currently provided across the UK. This will allow us to further improve access to care for all women across all regions.

We will therefore be extremely grateful in your support in distributing this questionnaire to the relevant people in the hospitals in your trust.

Please return to: Dr Melanie Nana Melanie.nana@gstt.nhs.uk

If you have any queries, please also contact Melanie Nana. Thanks in advance for your time.

Yours sincerely,

Professors Catherine Nelson-Piercy and Catherine Williamson

**Nausea and vomiting in pregnancy (NVP)/Hyperemesis Gravidarum (HG): a survey of UK practice**

Hospital name:

Trust:

Maternal medicine network (if known):

| **Details of hyperemesis service** | | **Response** | | |
| --- | --- | --- | --- | --- |
| 1 | Are your patients routinely offered screening for NVP/HG at their booking visit? | Yes  No | | |
| 2 | Do you offer community care for women with NVP/HG? (e.g. in a community day centre or at home) | Yes – in a community day centre  Yes – at home  Before COVID-19 – in a community day centre  Before COVID-19 – at home  No | | |
| 3 | Do you offer ambulatory management for women with NVP/HG? | Yes  No | | |
|  |  | If yes, where? | Early pregnancy unit  Emergency department  Acute medical unit Gynaecology ward  Obstetric unit  Other | |
|  |  | If ‘Other’ please specify: | | |
| 4 | If admitted to hospital in which locations are NVP/HG managed? | Gynaecology ward throughout entire gestation  Obstetric ward throughout entire gestation  Medical ward throughout entire gestation  Under the care of the obstetric medicine team  Different setting depending on gestation | | |
|  |  | If ‘Different setting depending on gestation’ please specify (*e.g. gynaecology ward <18 weeks, obstetric ward >18 weeks gestation):* | | |
| 5 | Which of the following criteria do you use for admission for inpatient management? Select all that apply. | Continued nausea and vomiting, inability to keep down oral antiemetics | |  |
|  |  | Continued nausea and vomiting associated with weight loss despite oral antiemetics | |  |
|  |  | Ketonuria | |  |
|  |  | Confirmed/suspected comorbidity (*e.g. urinary tract infection)* | |  |
|  |  | Other | |  |
|  |  | If ‘Other’ please specify: | | |

| **Assessment and management** | | | | | | | | |
| --- | --- | --- | --- | --- | --- | --- | --- | --- |
| **Which drugs/therapies are routinely recommended by your service?**  Please check the appropriate box | | | | | | | | |
| **Therapy** | | **As 1^st^ line medication** | **As 2^nd^ line medication** | **As 3^rd^ line medication** | **Only after 1^st^ trimester** | **For a maximum of 5 days** | **As required (PRN)** | |
| **Ginger** | |  |  |  |  |  |  | |
| **Acustimulations** | |  |  |  |  |  |  | |
| **Hypnosis** | |  |  |  |  |  |  | |
| **Ondansetron** | |  |  |  |  |  |  | |
| **Cyclizine** | |  |  |  |  |  |  | |
| **Domperidone** | |  |  |  |  |  |  | |
| **Prochlorperazine** | |  |  |  |  |  |  | |
| **Promethazine** | |  |  |  |  |  |  | |
| **Chlorpromazine** | |  |  |  |  |  |  | |
| **Metoclopramide** | |  |  |  |  |  |  | |
| **Thiamine** | |  |  |  |  |  |  | |
| **Pyridoxine** | |  |  |  |  |  |  | |
| **Corticosteroids** | |  |  |  |  |  |  | |
| **Diazepam** | |  |  |  |  |  |  | |
| **Proton pump inhibitor** | |  |  |  |  |  |  | |
|  | | | | | | | | |
| 7 | Do you require patients to sign a risk form when prescribed any of the above? | | | | Yes  No | | | |
|  |  |  |  |  | If ‘Yes’, please specify: | | | |
| 8 | Which IV rehydration do you routinely offer? Please select all: | | | | 0.9% Normal saline | | |  |
|  |  |  |  |  | Hartmann’s solution | | |  |
|  |  |  |  |  | Dextrose | | |  |
| 9 | Do you offer enteral or parenteral nutrition for patients resistant to treatment? | | | | Yes  No | | | |
| 10 | Are patients routinely offered a mental health screen? | | | | Yes  No | | | |

| **Pre-pregnancy counselling** | | **Response** |
| --- | --- | --- |
| 11 | Does your unit offer pre-pregnancy counselling for women with a history of severe NVP/HG? | Yes  No |
| 12 | Do you have any further comments regarding management of NVP/HG patients in your trust? | |
|  |  | |
