# Supplementary material for: How are hospitals in England, Scotland and Wales caring for women with nausea and vomiting in pregnancy: a national service evaluation
Source: BMC Health Serv Res. 2025 Aug 25;25:1128. doi: 10.1186/s12913-025-12909-0 (PMC12376445; doi:10.1186/s12913-025-12909-0)
Supplement: Supplementary file 2 — Supplementary Material 2. [file 12913_2025_12909_MOESM2_ESM.docx]

Supplementary Table 1: Details of community and ambulatory services available.

| **Community treatment available (n=129), n (%)**   - At home - In a community day centre | **19 (14.8%)**  15/19 (78.9%)  4/19 (21.1%) |
| --- | --- |
| **Ambulatory management available (n=129), n (%)**   - In an early pregnancy unit - On a gynaecology ward - On an obstetric unit - In a maternity assessment unit - On an acute medical ward - In the emergency department - On a surgical assessment unit - Other | **108 (84.4%)**  46/108 (42.6%)  24/108 (22.2%)  18/108 (16.7%)  10/108 (9.3%)  1/108 (0.9%)  5/108 (4.6%)  2/108 (1.9%)  2/108 (1.9%) |
